# Diagnostic and prognostic value of miR-106a in colorectal cancer

**DOI:** 10.18632/oncotarget.13766

**Published:** 2016-12-01

**Authors:** Haibin Hao, Laipeng Liu, Dong Zhang, Chao Wang, Guangfeng Xia, Fuping Zhong, Xiaoyun Hu

**Affiliations:** ^1^ Department of General Surgery, Second Affiliated Hospital of Nanchang University, Nanchang, China

**Keywords:** miR106a, colorectal cancer, CRC, diagnosis, prognosis

## Abstract

We sought to systematically evaluate the diagnostic and prognostic value miR106a in patients with colorectal cancer (CRC). An original study was conducted to explore correlations between tissue miR106a levels and outcomes for 138 patients diagnosed with CRC. To explore the diagnostic performance of miR106a, eligible studies were identified from medical databases from China and abroad. Based on these results, 15 studies (including our original study) were pooled and included in a meta-analyses. The pooled sensitivity, specificity, and diagnostic odds ratios of miR106a were 0.53 (95% confidence interval (CI): 0.49–0.57), 0.85 (95% CI: 0.82–0.88), and 7.22 (95% CI: 3.17–16.44) for diagnosis of CRC, and the area under the curve (AUC) for miR106a when diagnosing CRC was 0.72. Patients with higher expression of tissue miR106a had poor overall survival (pooled hazard ratio (HR): 1.50; 95% CI: 1.02–2.20), but not disease-free survival (pooled HR: 1.03; 95% CI: 0.40–2.65). Overexpression of miR106a may predict superior metastasis-free survival (pooled HR: 0.65; 95% CI: 0.33–1.27), but the effect was not significant in this study (*p* = 0.21).

## INTRODUCTION

CRC is one of the most common cancers and a leading cause of death worldwide [1]. Currently, surgery is the primary treatment, while supplementary treatments include chemotherapy and molecularly targeted therapy [1, 2]. Most patients are diagnosed with CRC at an advanced stage, leading to a low cure rate and poor prognosis [3]. More specific tumor markers could improve CRC diagnosis and treatment. microRNAs (miRNA) are 21-25nt single-stranded, non-coding RNAs, highly conserved in evolution, which regulate target gene expression by inhibiting mRNA translation or inducing degradation of the mRNA [4]. Calin and colleagues published the first study linking miRNAs to cancer [5]. Since that time, many studies have further suggested that miRNAs may provide a new method for cancer screening, diagnosis, prognosis, and prediction of response to chemotherapy. Recent studies have reported altered expression of many miRNAs in CRC tissues, including miR-135b, miR-133, miR-21, miR-203, miR-106a and so on [6–8]. miR106a is an oncogenic miRNA that modulates the expression of cancer-related genes, including RUNX3 [9], Twist1 [10], and pRB [11]. Interestingly, miR106a expression is elevated during tumor suppression and reduced in colorectal cancer tissues [12]. Furthermore, miR106a expression level appears correlated to patient survival.

Many studies have evaluated the value of miR106a as a diagnostic or prognostic marker for colorectal cancer with contradictory results. As a consequence, the aim of this study was to comprehensively explore the potential value of miR106a in colorectal cancer diagnosis and prognosis.

## RESULTS

### The original study

**Figure 1:**
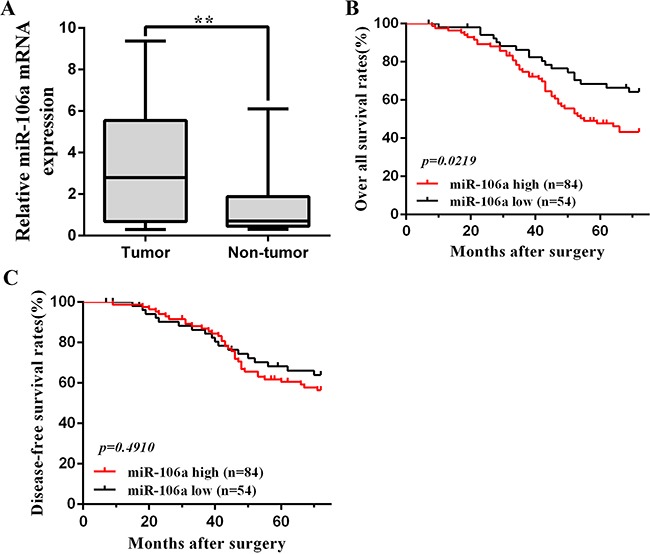
miR106a overexpression in CRC clinical samples **A**. Quantification of miR106a mRNA expression in CRC tissues and non-tumor tissues. **P<0.01, compared with non-tumor counterpart. **B**. Kaplan-Meier analysis of overall survival in 138 patients based on the tumor level of miR106a expression. The overall survival of the high miR106a patients(n=84) was significantly worse than that of the low miR106a patients (n=54), and *p*=0.0219. **C**. Kaplan-Meier analysis of disease-free survival in 138 patients. The prognostic value of miR106a is not significant (*p*=0.491). The log-rank test was used to calculate p-values.

We included 138 patients with CRC in the present original study to assess the prognostic value of tissue miR106a. miR106a expression was increased in CRC tumor relative to normal tissues with a median tumor to normal (T/N ratio) of 3.98 (Figure 1A), and each patient's detailed and specific miR106a expression is shown in Supplementary Figure S1. Association between miR106a expression and clinical characteristics of the series are listed in Table 1. Positive associations were found between expression of miR106a and tumor size (p=0.029), preoperative CEA level (p=0.013), and T classification (p=0.004) (Table 1). The mean follow-up time for all patients was 53.3 months (95% CI, 49.9–56.7), and 41 patients died of CRC during the follow-up period. Kaplan-Meier survival analysis revealed an association between OS (61%) and miR-106a expression (p=0.0219). Patients with low miR-106a levels (lower 2 tertiles of all patient samples) had an OS rate of 65% (95% CI; 48–82%), whereas patients with high levels (highest tertile) had a rate of 43% (95% CI; 28–58%) (Figure 1B). Univariate Cox proportional hazard regression analysis revealed an HR of 2.03 (95% CI, 1.05–3.95; P = 0.027) for tissue miR106a in CRC prognosis (Supplementary Table S2). In the multivariable analysis, which included miR106a level, age, side of the tumor, TNM stage, differentiation and so on, the HR for tissue miR106a in colorectal cancer prognosis was 1.87 (95% CI, 1.13–3.09; P = 0.03). miR106a expression did not affect the DFS (p=0.491) (Figure 1C). The univariate and multivariable analysis of DFS revealed HR of 0.83 (95% CI, 0.40, 1.72; P = 0.55) and 1.22 (95% CI, 0.70, 2.12; P = 0.43), respectively.

**Table 1:** Relationship of miR-106a level in cancer tissues and clinicopathological factors in patients with colorectal cancer

colorectal cancer	138 cases		miR-106a^a^
	n	%	
Sex			
Male	92	66.7	1.491(0.460,2.010)
Female	46	33.3	1.326(0.449,1.855)
*p*			0.887
Age (years)			
>60	67	48.6	1.434(0.514,1.381)
≤60	71	51.4	1.438(0.406,2.015)
*P*			0.486
Tumor size(cm)			
<5	60	43.5	1.015(0.479,1.707)
≥5	78	56.5	1.760(0.447,2.729)
*P*			0.029*
Preoperative CEA level			
<5 ng/mL	82	59.4	0.920(0.487,1.245)
≥5 ng/mL	56	40.6	2.192(0.417,3.986)
*P*			0.013*
Histologic grade			
well differentiated	102	73.9	1.401(0.438,2.011)
poorly differentiated	36	26.1	1.536(0.674,0.995)
*P*			0.356
T classification			
T1+T2	48	34.8	1.101(0.342,1.822)
T3+T4	90	65.2	1.615(0.523,2.034)
*P*			0.004*
TNM stage			
I	18	13	1.383(0.396,2.258)
II	31	22.5	1.044(0.338,1.706)
III	64	46.4	1.459(0.514,1.768)
IV	25	18.1	1.901(0.507,3.943)
*p*			0.101
Lymphatic invasion			
Negative	53	38.4	1.549(0.414,2.418)
Positive	85	61.6	1.365(0.481,1.699)
*p*			0.509
Distant metastasis			
Negative	104	75.4	1.395(0.448,1.822)
Positive	34	24.6	1.562(0.601,2.622)
*p*			0.401

^a^Mean of relative expression with 25th–75th percentile is recorded in parentheses, use nonparametric test (Mann–Whitney U test between two groups and Kruskal–Wallis H test for three or more groups); *p<0.05.

### Study selection and characteristics

**Figure 2:**
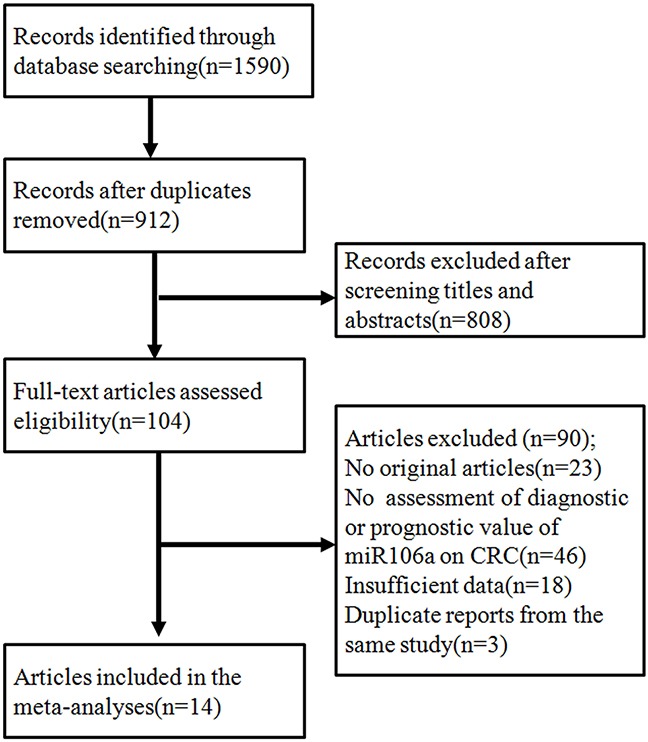
The flowchart showed the selection of studies for meta-analysis

After initial searches of PUBMED, EMBASE, CBM, CNKI and Wan Fang Data, 1590 articles were retrieved. A total of 14 articles were identified as eligible studies [12–25]. Including our original study, 15 studies were included in the meta-analyses. The selection process is shown in Figure 2, and the characteristics of the included studies are presented in Supplementary Tables S3 and S4. Among the included articles, 11 reported the prognostic value of miR106a (including our study) [12–16, 19, 20, 22, 23, 25], whereas 6 examined diagnostic value of miR106a [15, 17, 18, 20, 21, 24] (2 articles reported both prognostic and diagnostic value [15, 20]).

### Diagnostic value of miR106a for CRC

**Figure 3:**
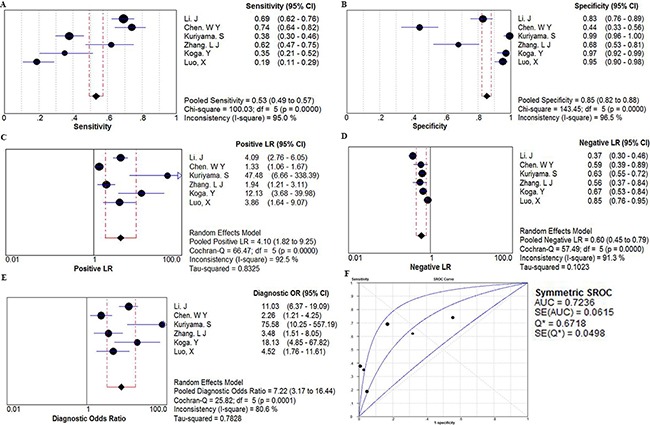
The forest plots show the pooled diagnosis index and Summary receiver operating characteristic curves (SROC) of miR-106a for the diagnosis of CRC The point efficiencies from each study are shown as squares and the pooled efficiencies are shown as diamond. Degree of freedom is abbreviated as df. Inconsistency is used to quantify the heterogeneity caused by non-threshold effect. Of these studies, random effects model was used to pool these data. **A**. sensitivity; **B**. specificity; **C**. positive likelihood ratio(PLR); **D**. negative likelihood ratio(NLR); **E**. diagnostic OR(DOR), and their 95% CI are displayed respectively, which suggests miR106aa might be a potential noninvasive diagnosis biomarker of CRC. **F**. Every square stands for a study. The SROC curve is symmetric and the AUC is 0.7236, which intimates a moderate diagnostic accuracy for diagnosing CRC.

Six studies with 1213 total patients assessed the diagnostic value of miR106a level for CRC. Sample size of each study ranged from 97 to 305. The types of specimen included serum (n = 1), plasma (n =3), and fecal (n=2). All studies used quantitative reverse transcription PCR (qRT-PCR) to measure the expression of miR106a. The quality assessments are shown in Supplementary Figure S2A. In order to assess the heterogeneity of miR106a among the eligible studies, we first calculated the correlation coefficient and p value between the logit of sensitivity and logit of 1-specificity using Spearman test to exclude the threshold effect. The resulting Spearman correlation coefficient was 0.714 and the p value was 0.111, indicating that there was no heterogeneity from threshold effect. Because of potential heterogeneity caused by non-threshold effect among these studies, the random effect model was used to estimate overall performance of miR106a. For miR106a, the sensitivity, specificity, positive likelihood ratio (PLR), negative likelihood ratio (NLR), and DOR of 6 included studies were evaluated by forest plots (Figure 3). The pooled sensitivity and specificity were 0.53 (95% CI, 0.49–0.57) and 0.85 (95% CI, 0.82–0.88), respectively (Figure 3A and 3B). PLR and NLR were 4.10 (95% CI: 1.82–9.25) and 0.60 (95% CI: 0.45-0.79) (Figure 3C and 3D). The summary DOR (Figure 3E) and the area under SROC (Figure 3F) were 7.22 (95% CI: 3.17–16.44) and 0.72, indicating miR106a has a relatively high diagnostic performance in CRC.

### The prognostic meta-analyses

**Figure 4:**
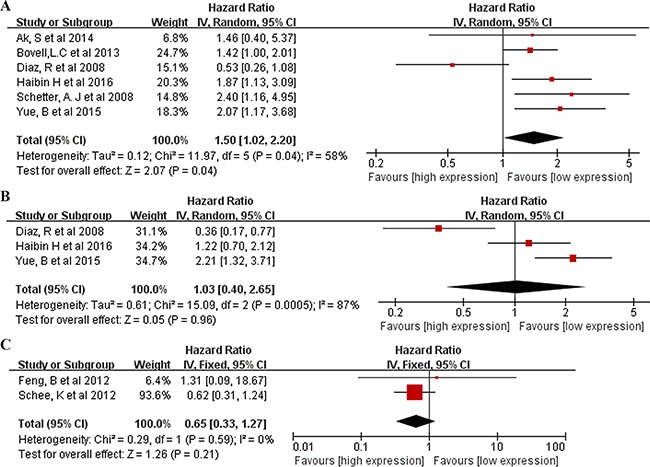
Forrest plots of studies evaluating hazard ratios of high miR106a level **A**. Overall survival test. The survival data from 6 articles were pooled to calculate overall survival. The random effects analysis model showed the pooled HR for overall survival is 1.50 with 95% CI:1.02–2.20, and P <0.05. **B**. Survival data were presented as disease-free survival, the random effect analysis model was used to calculate the pooled HR, and HR = 1.03 (95%CI: 0.40–2.65, P >0.05) for DFS. **C**. Survival data were presented as metastasis-free survival, the fixed effect analysis model was used to calculate the pooled HR, and HR = 0.65 (95%CI: 0.33–1.27, P >0.05) for MFS.

A total of 11 studies were included in the prognostic analyses (Supplementary Table S4). All were published in English and conducted in Europe (n = 4), East Asia (n = 5), or the United States (n = 2; one study included both American and Chinese populations). Each study assessed 40 to 345 patients with CRC. The types of specimen included solid tissue (n = 8) and serum (n = 3) (Supplementary Table S5). All studies used qRT-PCR to measure miR106a expression. The quality assessments are shown in Supplementary Table S4. Six studies (including our own) with 900 total patients assessed the relationship between tissue miR106a expression and CRC OS. The pooled HR was 1.50 (95% CI: 1.02–2.20) for all studies, indicating that higher tissue miR106a expression levels predicate poorer OS (p=0.04) (Figure 4A). Significant heterogeneity across studies was observed (I^2^ = 58%, p < 0.05; Figure 4A). Three studies comprising 335 patients evaluated CRC DFS for miR106a, and two studies evaluated MFS for miR106a. We found a nonsignificant association between miR106a expression level and DFS (pooled HR, 1.03; 95% CI, 0.40–2.65; Figure 4B). There was significant heterogeneity in the analysis for DFS (I^2^=87%, p< 0.05; Figure 4B) but not MFS (p> 0.05; Figure 4C). In addition, three studies explored the performance of circulating miR106a levels in the prognosis of colorectal cancer. The HRs of two of these studies for OS were 1.17 (95% CI, 0.90–1.52) and 1.80 (95% CI, 1.11–2.91), and the HR of one study for DFS was 3.02 (95% CI, 1.36–6.72) (Supplementary Table S5).

### Publication bias

The overall distribution of studies is summarized in the likelihood matrix in Supplementary Figure S2C, and the Fagan's nomogram describes how to use diagnostic finding from miRNA106a assay to calculate post-test cancer probability (Supplementary Figure S2D) [26]. For OS, Metainf investigated the influence of each study on the overall meta-analysis summary estimate, and Supplementary Figure S3B shows that the results of the meta-analysis did not change after the removal of any one paper [27]. The funnel plots are shown in. Funnel plot tests of the diagnostic and prognostic meta-analyses indicated no significant publication bias in this study (Supplementary Figure S2B and Supplementary Figure S3A). The Deeks funnel plot test for diagnostic value gave a p value of 0.31, and Begg and Egger tests for prognostic value provided p values of 0.71 and 0.90, respectively. However, because of the limited number of included studies, publication bias cannot be ruled out.

## DISCUSSION

Although significant progress has been achieved in the diagnosis and prognosis of CRC over the years, development of better biomarkers is still necessary for early detection and for predicting patient outcomes [28]. The application of miRNAs as biomarkers for cancer diagnosis and prognosis has gained much attention in recent years [29, 30]. miR106a is one of the most studied miRNAs as a potential biomarker of CRC diagnosis and prognosis [6]. To examine the reported diagnostic and prognostic accuracies, we performed this systematic review on 14 diagnostic or prognostic studies.

In the present meta-analysis, miR106a had a pooled sensitivity of 0.53, specificity of 0.85, and AUC of 0.72, suggesting that measuring miR106a level is a promising noninvasive method for CRC diagnosis. DOR combines the strengths of both sensitivity and specificity, and was reported to be a useful indicator for evaluation of the diagnostic method. The DOR value of miR106a was 7.22, indicating a moderate diagnostic accuracy. However, the positive LR (4.10) and negative LR (0.60) suggest that miR106a may not be adequate to distinguish patients with CRC. We found significant heterogeneity in sensitivity, different cutoff values of miR106a expression across studies may be one source of heterogeneity. Measuring circulating miR106a might also be a useful screening method for colorectal advanced adenoma. In a study of the Japanese population conducted by Kuriyama and colleagues, in which 138 patients with advanced adenoma and 126 control subjects were enrolled, miR106a had a relatively high diagnostic performance for advanced adenoma (AUC value of 0.826, sensitivity of 0.377, and a specificity of 0.992). In another study, which included 100 Chinese advanced adenoma patients and 79 healthy controls, miR106a yielded an AUC value of 0.605 for discriminating advanced adenomas from controls. It should be noted that these studies were both conducted in East Asia and the sample sizes were not large; thus, more studies are warranted to clarify this issue. On the other hand, the meta-analyses indicated that tissue miR106a expression level was a promising biomarker to predict survival in patients with CRC. Compared with patients with low tissue miR106a expression level, patients with an increased level of miR106a expression had a 1.50-fold higher risk of poor OS and 1.03-fold lower rate of DFS. There was significant heterogeneity in the meta-analyses of the data for OS and DFS. Our results showed that the effect of miR106a in predicting CRC survival was observed only in male participants, suggesting that gender may modify the observed effect. More large, well-designed studies are warranted to clarify this issue and explore the relevant mechanisms. Whether the prognostic value of other miRNAs differs by gender may also need further study. Circulating miR106a was also developed as a noninvasive prognostic biomarker for CRC, and studies indicated that higher circulating miR106a level might be associated with poor OS for CRC.

Though sensitivity and subgroup analyses were applied, heterogeneity in both diagnostic and prognostic meta-analyses was not fully explained. The heterogeneity might result from the different evaluating methods for miR-106a. Different kinds of samples are used in assessing miR106a expression, including tissues, serum, plasma and fecal. Normalization is another problem for quantitative estimation of miRNAs. For the included studies, RNU6B, miR16, and total RNA were used by different studies. However, To draw a convincing conclusion on the value of miR106a for the diagnosis and prognosis of CRC, an appropriate and unified method should be established and applied.

It is hypothesized that miRNAs enter the circulation directly secreted by cells, released by cells via exosomes, and via shedding of micro vesicles [31]. miRNAs demonstrate the same change in expression in plasma, serum, feces, and tumor tissues of patients with various types of cancer [32, 33]. Studies in human cell lines further investigated the physiologic targets of miR106a, and showed that miR106a could target tumor-suppressor genes, such as RUNX3, Twist1, pRB and E2F1 [34]. Therefore, miR106a may be involved in the critical steps in carcinogenesis and progression of human cancer by promoting tumor growth, proliferation, anti-apoptotic mechanisms, and migration. It has been further demonstrated that tissue miR106a expression is associated with lymph node positivity and the development of distant metastases for CRC; therefore miR106a expression serves as a clinicopathologic feature of the disease. These findings support a vital role for altered miR106a expression in tumorigenesis.

This systematic review had several important strengths. First, we conducted a relatively thorough systematic search and applied a comprehensive analytic approach to evaluate the diagnostic and prognostic value of miR106a in patients with CRC. Second, an original study was also conducted to explore prognostic potential of miR106a in CRC. The methods of this study were rigorous and followed the guidelines for conducting and reporting systematic reviews. There were also some limitations in our analysis. First, most of the diagnostic studies enrolled healthy people as controls and were not designed to be blind, which may affect diagnostic performance. Second, there was considerable heterogeneity for both the diagnostic and prognostic meta-analyses. Subgroup and sensitivity analyses were applied, but the results could not fully explain the observed heterogeneity. Third, the different chemical assays used in the included studies might result in systematic errors among studies.

Taken together, we conclude that miR106a level is a useful biomarker for CRC detection, and tissue miR106a is a promising marker for CRC prognosis. Further research is needed to explore the combination of other variables associated with CRC diagnosis and prognosis, in an effort to develop better diagnostic and prognostic models with higher discriminative capacity.

## MATERIALS AND METHODS

### Original study

CRC tissues and corresponding normal tissues were obtained from 138 patients by surgical resection. Total RNA was extracted from tissue samples, followed with DNase I digestion to exclude genomic DNA contamination. Mature miR106a and internal control U6 were detected by stem-loop real-time RT-PCR methods. Survival analyses were conducted using the Kaplan–Meier method. Univariate and multivariate Cox's proportional hazard regression analyses were applied to estimate HRs of death according to tissue miR106a expression levels. The detailed methods are described in the Supplementary Materials. The prognostic data calculated from the original study were pooled with studies identified from literature search in the meta-analysis process.

### Meta-analysis

This meta-analysis was designed, conducted, and reported according to the PRISMA statement [35]. The meta-analyses process was carried out in accordance with the Cochrane Handbook for Systematic Reviews of Intervention [36]. The review has been registered in an international registry of systematic reviews PROSPERO (CRD42013005119).

### Literature search and study selection

Comprehensive literature searches were conducted (up to December, 2015) in PUBMED, EMBASE, Chinese Biomedical Literature Database (CBM), Chinese National Knowledge Infrastructure (CNKI), and Wan Fang Data to identify eligible studies. The detailed selection process was presented in Supplementary Materials.

### Data extraction

Three reviewers independently collected data using standardized forms and discrepancies were resolved by a fourth investigator. The following information from each study was extracted: first author, year of publication, origin of the study population, patient characteristics (age, sex, cancer type, and stage), source of samples, number of participants, miR106a assay method, follow-up time, and variables adjusted for in the analysis. For diagnostic studies, the numbers of true-positive (TP), false-positive (FP), true-negative (TN), and false-negative (FN) results were extracted. For prognostic studies, HR estimates with 95% confidence intervals (CI) for overall survival (OS), disease-free survival (DFS), progression-free survival, or metastasis-free survival (MFS) were extracted. If the HRs and their 95% CIs were not provided, the numbers of deaths or recurrences and total samples in each study were extracted to calculate these numbers.

### Quality assessment

The quality of each diagnostic study was assessed independently by three investigators according to the QUADAS-2 (Quality Assessment of Diagnostic Accuracy Studies 2) [37]. The QUADAS-2 is recognized as an improved, redesigned tool which comprises 4 key domains (patient selection, index test, reference standard, and flow and timing) supported by signaling questions to aid judgment on risk of bias, rating risk of bias and concerns about applicability as “high”, “unclear” and “low,” and handling studies in which the reference standard consists of follow-up (Supplementary Table S1). For prognostic studies, Newcastle-Ottawa scale (NOS) was applied to assess the risk of bias and the criteria for reporting observational studies to complete the methodologic evaluation [38]. These scales were used to allocate a maximum of nine stars for quality of selection, comparability, exposure, and outcome of study participants. Studies with six or more stars are rated as high quality.

### Statistical analysis

All statistical analyses were performed by Meta-DiSc, Review Manager 5.2, STATA 12.0 and SPSS 22.0 statistical software. All accuracy data from each study (true positives (TP), false positives (FP), true negatives (TN), and false negatives (FN)) were extracted to obtain pooled sensitivity, specificity, positive likelihood ratio (PLR), negative likelihood ratio (NLR), positive predicted value, negative predicted value, diagnostic odds ratio (DOR) and their 95% confidence interval (95% CI), simultaneously, generate the summary receiver operator characteristic (SROC) curve and calculate the area under the curve (AUC)[39]. The sensitivity, specificity, positive and negative predicted value, diagnostic odds ratio of miR106a were presented as forest plots. Moreover, the heterogeneity between the studies caused by threshold effect was quantified using Spearman correlation analysis [40]. The Non-threshold effect was assessed by the Cochran-Q method and the test of inconsistency index (I^2^), and a low p value (≤0.05) and high I^2^ value (≥50%) suggest presence of heterogeneity caused by non-threshold effect. If the non-threshold effect existed, meta-regression would be used to find out the sources. The Deeks’ funnel plot method was applied to for test publication bias [41].

HR was adopted for prognostic evaluation in the current meta-analysis, because all of the included studies used HR to measure the prognostic performance of miR106a. Study-specific HR estimates were pooled using a fixed effects model, if there was no significant heterogeneity. Otherwise, a random-effects model was applied. The extent of heterogeneity across studies was checked using the χ^2^ and I^2^ tests; P 0.10 and/or I^2^ > 50% indicates significant heterogeneity [42]. Begg funnel plots and Egger linear regression test were used to assess publication bias [43, 44]. Tissue miR106a expression values were divided with the highest tertile classified as high and the lower two tertiles defined as low. Survival analyses were conducted using the Kaplan–Meier method. Then, univariate Cox's proportional hazard regression analyses were applied to estimate HR of death according to tissue miR106a expression levels. Multivariate models were used to adjust potential confounding factors for death, including age, sex, TNM stage, pathologic differentiation, and side of the tumor (left or right). Publication bias and sensitivity analysis were conducted using Stata 12.0. A p value of <0.05 was considered statistically significant.

### Ethics statement

The study has been approved by the Ethics Committee of our institution that conforming to the provisions of the Declaration of Helsinki.

## SUPPLEMENTARY MATERIALS FIGURES AND TABLES






