# Supplementary material for: Diagnostic and prognostic value of miR-106a in colorectal cancer
Source: Oncotarget. 2016 Dec 1;8(3):5038–47. doi: 10.18632/oncotarget.13766 (PMC5354890; doi:10.18632/oncotarget.13766)
Supplement: Supplementary file 1 [file oncotarget-08-5038-s001.pdf]

Diagnostic and prognostic value of miR-106a in colorectal cancer

SUPPLEMENTARY DATA

SUPPLEMENTARY METHODS FOR ORIGINAL STUDY

Tissue processing

This study was approved by the local Human Investigations Committees of the Second Affiliated Hospital of Nanchang University, Nanchang, and all patients were enrolled with informed consent under institutional review board-approved protocols. CRC tissue and corresponding normal tissues were obtained from 138 patients by surgical resection in the our hospital between January 2008 and October 2015. Normal tissues were validated by histopathology, and were taken from the surgical margins, at least 10 cm away from tumor. After these procedures, all tissues samples were immediately frozen in liquid nitrogen and kept at -80˚C until RNA extraction. No patients had received adjuvant treatment including radiotherapy or chemotherapy prior to surgery and diagnosis.

RNA isolation and qRT-PCR

Total RNA was extracted from tissue and plasma samples using Mini RNeasy Kits for tissues (QIAGEN, CA, USA) and TRIzol LS Reagent (Invitrogen, CA, USA) for plasma, followed with DNase I digestion using the RNase-Free DNase Set (QIAGEN, CA, USA) to exclude genomic DNA contamination. Mature miR106a and internal control U6 were detected by stem-loop real-time RT-PCR analysis using Taman Human MicroRNA Assay kits (Applied Biosystems, Foster City, CA, USA).

The specific primer of miR106a and U6 is used (5'-GGAAAAGTGCTTACAGTGCAGGTAG-3';5'-CGC TTCGGCAGCACATATAC-3'). Relative expression values were calculated using the comparative CT method and by normalizing with U6 as an endogenous control. Fold change $=2^{-\Delta\Delta C_T}$, where $\Delta\Delta C_T = \Delta C_T(C_{T\mathrm{miRNA}}\text{-}C_{TU6})_{\mathrm{tumor}} - \Delta C_T(C_{T\mathrm{miRNA}}\text{-}C_{TU6})_{\mathrm{normal}}$.

Heat map for gene differential expression

Heat map analysis, also known as cluster analysis was used to judge clustering mode under different experimental conditions by R-heatmap.2 (http://www.inside-r.org/packages/cran/gplots/docs/heatmap.2). To classify different expression by hierarchical clustering or K-means and other methods, and different colors represent different cases of the cluster group. In my heat map, all fold-change calculations from CT values using the ΔΔCT method of relative quantification were input, minValue and maxValue were found. Gradient legend is a pane of blended colors derived from the color range definitions. A linear scale is drawn with two drag-able pointers. Each color defined for a numeric range blends with the next color, thus forming a gradient strip.

Statistical analysis

To determine the diagnostic performance of serum miR106a level in colorectal cancer, receiver operating characteristic (ROC) analysis was performed and the area under the curve (AUC) value was calculated. The optimal cutoff threshold was determined at the point on the ROC curve at which (sensitivity+apecificity-100%) was maximal. Sensitivity and specificity were calculated with this cutoff value.

Survival analyses were conducted using the Kaplan-Meier method. Univariate Cox's proportional hazard regression analyses were applied to estimate hazard ratios (HRs) of death according to tissue miR106a expression levels. And multivariate models were used to adjust potential confounding factors for death, including age, sex, TNM stage, pathological differentiation and side of the tumor (left or right).

All analyses were conducted using the Stata software (version 12.0; StataCorp, College Station, TX, USA), Meta-DiSc(version 1.4; Clinical Biostatistics Unit, Ramón y Cajal Hospital, Spain), Review Manager 5.2 (the Nordic Cochrane Center, Rigshospitalet, Denmark) and SPSS 11 (Chicago, IL, USA). $P<0.05$ was considered statistically significant.

Supplementary methods for literature search and study selection

The following keywords were used in the searches: ("colorectal" OR "colon" OR "rectal") AND ("cancer" OR "tumor" OR "carcinoma" OR "adenocarcinoma") AND ("micro RNA" OR "micro rna" OR "miR" OR "micro RNA106a"OR "miR106a"). References of relevant articles and reviews were also scanned to include possible missed articles. Titles and abstracts were first scanned, and then full papers of potential eligible studies were

reviewed. Meeting abstracts were excluded because of the limited data. Articles as full papers in English and Chinese were evaluated for eligibility. The retrieved studies were carefully examined to exclude potential duplicates or overlapping data.

Articles were included if they met all the following criteria: (1) study should evaluate diagnostic or prognostic value of blood or tissue miR106a level in CRC patients. (2) for diagnostic studies, histologic assessment should be applied as reference standard for CRC; and (3) for studies analyzed the diagnostic value of miR106a, absolute number of true-positive (TP), false-positive (FP), true-negative (TN) and false-negative (FN) were reported or could be calculated; for prognostic studies, hazard ratio (HR) or risk ratio (RR) values with 95% confidence intervals (95% CI) were provided or could be calculated.

SUPPLEMENTARY FIGURES

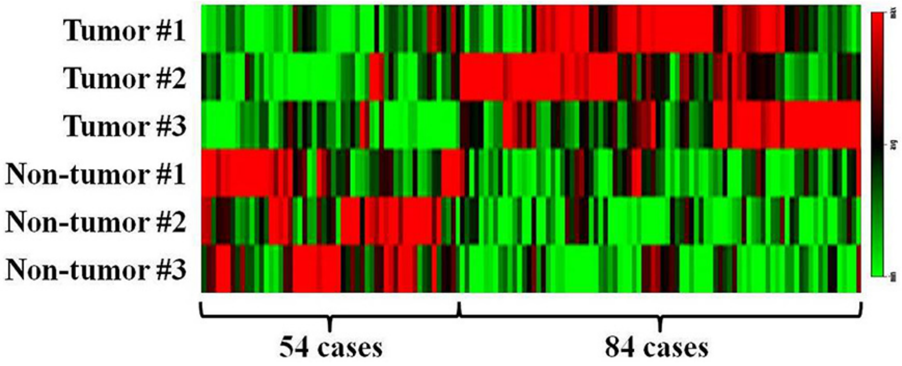

Supplementary Figure S1: Heat map of 138 clinical tissues in tumor and non-tumor reflected miR106a mRNA expression values (n=3, each tissue). The picture shows the overall hierarchical clustering map, $2^{-\Delta\Delta CT}$ value as cluster. Red indicates high expression of the gene, green indicates low expression. The x-axis represents different samples, y-axis indicates tumor or non-tumor. The color key at the right of map represents the range which was from min to max.

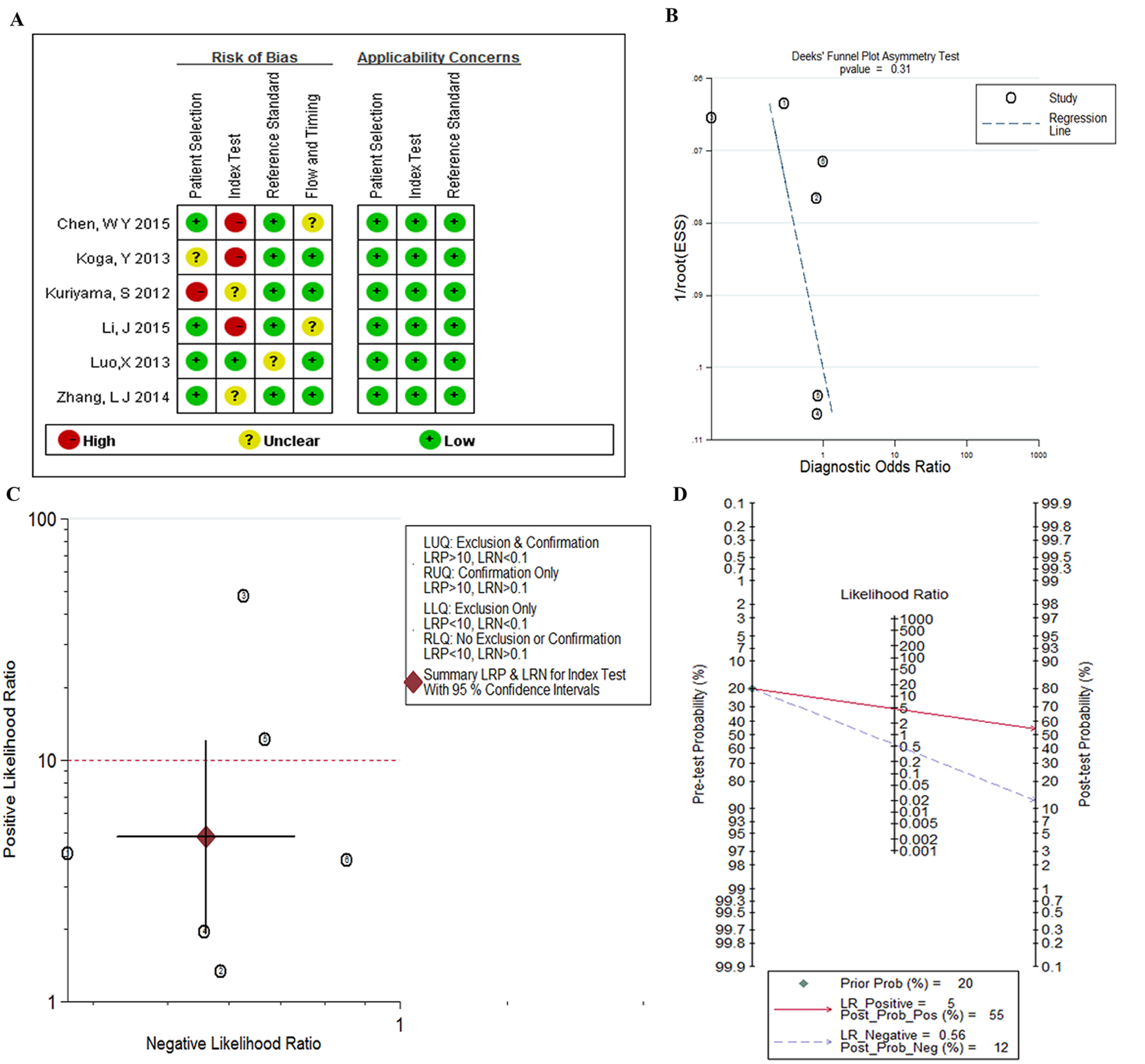

Supplementary Figure S2: The quality assessment and publication bias based on the eligible studies for diagnosis. A. Quality assessment of the included studies by QUADAS-2. It summarized “risk of bias” and “applicability concerns” through judging each domain for each included study. It shows the major biases concentrated upon the “patient selection” and “index text”. **B.** Publication bias from Deeks’ test is shown by funnel plots. Every point represents one study and the line is the regression line. It shows no publication bias exists. **C.** The overall distribution of studies is summarized in the likelihood matrix. Each point corresponds to a study. One studies, Li. J et al was on the left side of the matrix, indicating a sensitive “rule out” test. However, it reported reasonable sensitivity with incorporation bias from knowledge of a desaturation study outcome. **D.** Fagan’s nomogram describes the possibility miR-106 assay to confirm or exclude cancer patients. In detail, for any people with a pre-test probability of 20% to have cancers, if the miR-106a test in cancer detection was positive, the post-test probability to have cancer would rise to 55%; while a negative result of miR-106 assay meaning the post-test probability would drop to 12% for the same people. Hence, miR-106a assay may play an important role as initial screening method for cancer.

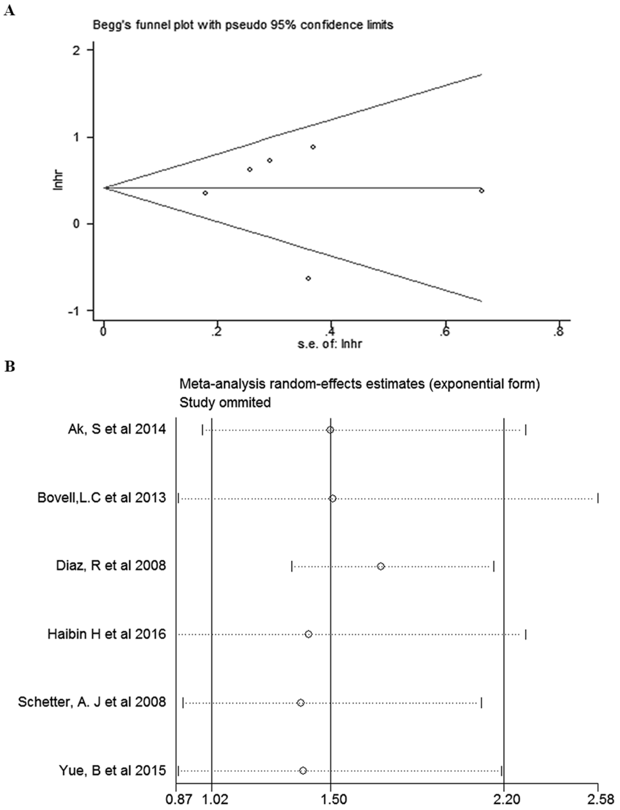

Supplementary Figure S3: The publication bias and sensitivity analysis based on the studies for prognosis of OS. A. Publication bias from Beeg' test is shown by funnel plots. Every point represents one study. **B.** Forest plot for the sensitivity analysis, shows the results of the meta-analysis did not change after the removal of any one paper.

SUPPLEMENTARY TABLES

Supplementary Table S1: Instrument used for the evaluation of the risk of bias and applicability concerns of the included prognostic studies (adapted from QUADAS-2)

See Supplementary File 1

Supplementary Table S2: Adjusted hazard ratio (95% CI) of survival using a cox proportional hazard regression model

	Univariate		Multivariate	
	**HR**	**(95% CI)**	**HR**	**(95% CI)**
**OS**	2.03	1.05-3.95	1.87	1.13-3.09
**DFS**	1.31	0.68-2.32	1.22	0.70-2.12

HR: hazard ratio, 95%CI: confidence interval

Supplementary Table S3: Main characteristics of studies include in meta-analysis for diagnosis

First author (year)	Country	Patients/ controls	Mean or medianAge (years)	Assay type	Internalcontrol	Cut-offvalues	SE[a]	SP[b]	AUC[c]
Chen, W Y(2015)	China	100/79	59.5	qRT-PCR,$2^{-\Delta\Delta ct}$	miR-16	2.03	0.74	0.444	0.605
Koga, Y(2013)	Japan	40/104	62.6	qRT-PCR,$2^{-\Delta\Delta ct}$	miR-24	0.43	0.342	0.972	-
Kuriyama, S(2012)	Japan	138/126	-	qRT-PCR,$2^{-\Delta\Delta ct}$	miR-16	-	0.377	0.992	0.826
Li, J (2015)	China	175/130	56.1	qRT-PCR,$2^{-\Delta\Delta ct}$	let-7d/g/i	1.613	0.785	0.828	0.813
Zhang, L J(2014)	China	50/47	59.5	qRT-PCR,$2^{-\Delta\Delta ct}$	miR-16	-	0.623	0.682	0.661
Luo, X (2013)	Germany	80/144	58	qRT-PCR,2-ΔΔct	miR-16	-	0.19	0.95	-

[a]: sensitivity. [b]: specificity. [c]: The area under the curve.

Supplementary Table S4: Summary table of main characteristics for the eligible studies

See Supplementary File 2

Supplementary Table S5: Summary table of HRs and their 95%CI

Study(year)	HR	95%CI(LL-UL)	p-value[a]	outcome[b]	origin
Ak, S et al 2014	1.46	0.4-5.37	0.567	OS	tissue
Bovell, L.C et al 2013	1.42	1-2.01	0.005	OS	tissue
Chen, W. Y et al 2015	1.8	1.11-2.91	0.02	OS	blood
Diaz, R et al 2008	0.36	0.17-0.77	0.009	DFS	tissue
Diaz, R et al 2008	0.53	0.26-1.08	0.07	OS	tissue
Feng, B et al 2012	1.31	0.09-18.67	<0.05	MFS	tissue
Kjersem, J.B et al 2014	1.17	0.9-1.52	0.231	OS	blood
Haibin H et al 2016	1.87	1.13-3.09	0.0219	OS	tissue
Haibin H et al 2016	1.22	0.70-2.12	0.491	DFS	tissue
Li, J et al 2015	3.02	1.36-6.72	0.007	DFS	blood
Schee, K et al 2012	0.62	0.31-1.24	0.56	MFS	tissue
Schetter, A. J et al 2008	2.4	1.16-4.95	0.02	OS	tissue
Yue, B et al 2015	2.21	1.32-3.71	0.034	DFS	tissue
Yue, B et al 2015	2.07	1.17-3.68	0.073	OS	tissue

[a]: Reported in the article, [b]: DFS already includes PFS, MFS.
