# Supplementary material for: Diagnostic and prognostic value of miR-106a in colorectal cancer
Source: Oncotarget. 2016 Dec 1;8(3):5038–47. doi: 10.18632/oncotarget.13766 (PMC5354890; doi:10.18632/oncotarget.13766)
Supplement: Supplementary file 2 [file oncotarget-08-5038-s002.doc]

**Supplementary Table 1** Instrument used for the evaluation of the risk of bias and applicability concerns of the included prognostic studies (adapted from QUADAS-2)

| **Domain** | **Patient Selection** | **Prognostic markers** | **Outcome** | **Flow** | **Timing** | **Confounding** |
| --- | --- | --- | --- | --- | --- | --- |
| Description | Describe methods of  patient selection  Describe included  patients (previous  testing, presentation,  intended use of index  test, and setting) | Describe the  prognostic marker and  how it was conducted  and interpreted | Describe the outcome  measurement and how  it was conducted and  interpreted | Describe any patients  who did not had the  prognostic markers  measured or for whom  outcome was not  measured or who were  excluded from the 2 X  2 table (refer to flow  diagram) | Describe the interval  and any interventions  between prognostic  markers and the  outcome  measurement | Describe any method  used to control for  potential confounding |
| Signaling  questions (yes,  no, or unclear) | Was a consecutive or  random sample of  patients enrolled?  Was a case–control  design avoided?  Did the study avoid  inappropriate  exclusions? | Were the prognostic  marker results  interpreted without  knowledge of clinical  data? (blinding)  If a threshold was  used, was it  prespecified? | Was the outcome measurement  adequate? Did all patients had  their outcome evaluated the same  way? Were the outcome  results interpreted  without knowledge of  the results of the  prognostic markers?  (blinding) | Did all patients have  outcome measured?  Were all patients  included in the  analysis?  (lost to follow-up?  withdrawal? patients  not tested? missing  data? etc.) | Was there an  appropriate interval  between prognostic  marker and outcome  measurement?  (sufficient time for  outcome to occur?) | Did the study  controlled for  potential  confounders? |
| Risk of bias (high,  low, or unclear) | Could the selection of  patients have  introduced bias? | Could the conduct or  interpretation of the  prognostic marker  have introduced bias? | Could the outcome  measurement, its  conduct, or its  interpretation have  introduced bias? | Could the patient flow  have introduced bias? | Could the timing have  introduced bias? | Confusion bias? |
| Concerns about  applicability (high,  low, or unclear) | Are there concerns  that the included  patients do not match  the review question? | Are there concerns  that the prognostic  marker, its conduct, or  its interpretation differ  from the review  question? | Are there concerns  that the outcome  measure does not  match the review  question?  (not clearly reported  according to prognostic  marker results or  different  dichotomisation of scales, etc.) |  |  |  |
