# Supplementary material for: Diagnostic and prognostic value of miR-106a in colorectal cancer
Source: Oncotarget. 2016 Dec 1;8(3):5038–47. doi: 10.18632/oncotarget.13766 (PMC5354890; doi:10.18632/oncotarget.13766)
Supplement: Supplementary file 3 [file oncotarget-08-5038-s003.doc]

**Supplementary Table 4**.Summary table of main characteristics for the eligible studies

| **Study(year)** | **origin of population** | **study design** | **NO. of patients(M/F)** | **Age(y)** | **Stage** | **miR-106a assay** | **cut-off** | **Survival analysis** | **follow-up months** | **Hazard Ratios** | **MSQA** |
| --- | --- | --- | --- | --- | --- | --- | --- | --- | --- | --- | --- |
| Ak, S et al 2014 | Turkey | R | 40(17/23) | 36.67 | I–IV | qRT-PCR | median normal tissue expression value | OS | 24–200 | SC | 8.7 |
| Bovell,L.C et al 2013 | USA | R | 345(170/175) | – | I–IV | qRT-PCR | median | OS | blacks 228 (84–377) , whites 180(84–360) | DE | 8 |
| Chen, W. Y et al 2015 | China | R | 100(60/40) | 58.91 | – | qRT-PCR | median | OS | – | reported | 7 |
| Haibin H et al 2016 | China | R | 138(92/46) | 56.3 | I–IV | qRT-PCR | mean normal tissue expression value | OS/DFS | 72 | SC | 8.7 |
| Diaz, R et al 2008 | Spain | R | 110(71/39) | 69 | I–IV | qRT-PCR | median | OS/DFS | 68–99 | reported | 9 |
| Feng, B et al 2012 | China | R | 28(15/13) | – | – | qRT-PCR | median normal tissue expression value | MFS | <60 | SC | 7 |
| Kjersem, J.B et al 2014 | Norway | R | 60(31/29) | 61 | – | qRT-PCR | median | OS | – | reported | 7.7 |
| Li, J et al 2015 | China | R | 175(113/62) | 57.6 | II/III | qRT-PCR | median | DFS | 27.5-38.1 | reported | 7.7 |
| Schee, K et al 2012 | Norway | R | 193(112/81) | – | I–III | qRT-PCR | median | MFS | – | SC | 8 |
| Schetter, A. J et al 2008 | USA/China | R | 197(122/75) | 59.6 | I–IV | qRT-PCR | median | OS | 24-147.2 | reported | 8 |
| Yue, B et al 2015 | China | R | 70(42/28) | – | I–IV | qRT-PCR | median | OS/DFS | – | reported | 6.7 |

Study design is described as consecutive patients (**C**), prospective (**P**) or retrospective (**R**).**OS**:overall survival,**DFS**:disease-free survival,**MFS:**metastasis-free survival,**PFS**: progress-free survival **DE**:data extrapolated,**SC**:survival curve;**MSQA**:Mean score of quality assessment.
